# Linear-Scaling Quantum Circuits for Computational Chemistry

**DOI:** 10.1021/acs.jctc.3c00376

**Published:** 2023-07-06

**Authors:** Ilias Magoulas, Francesco A. Evangelista

**Affiliations:** Department of Chemistry and Cherry Emerson Center for Scientific Computation, Emory University, Atlanta, Georgia 30322, United States

## Abstract

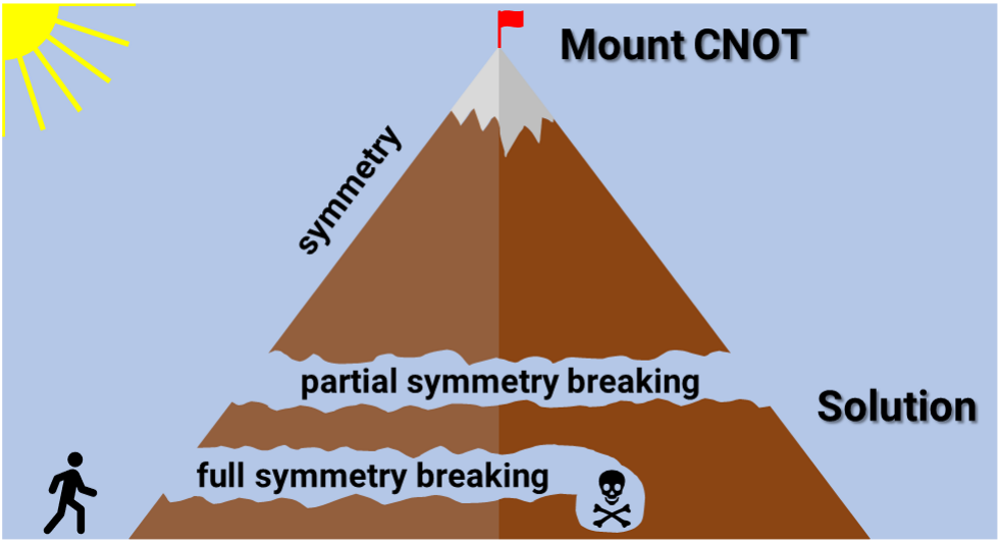

We have recently constructed compact, CNOT-efficient, quantum circuits for Fermionic and qubit excitations of arbitrary many-body rank [Magoulas, I.; Evangelista, F. A. J. Chem. Theory Comput.2023, 19, 8223665664310.1021/acs.jctc.2c01016]. Here, we present approximations of these circuits that substantially reduce the CNOT counts even further. Our preliminary numerical data, using the selected projective quantum eigensolver approach, show up to a 4-fold reduction in CNOTs. At the same time, there is practically no loss of accuracy in the energies compared to the parent implementation, while the ensuing symmetry breaking is essentially negligible.

Chemistry has been identified as one of the first potential killer applications for quantum computing.^1^ This is due to the fact that a quantum device can simulate a chemical problem with a number of computer elements (qubits) that scale, in principle, linearly rather than exponentially with system size. Even if an exponential advantage cannot be achieved for every chemical problem of interest,^2^ any form of polynomial speed up could potentially bring classically intractable applications within computational reach.

Several low-depth hybrid quantum–classical approaches have been proposed that are suitable for the current noisy intermediate-scale quantum hardware. In general, they can be divided into two broad categories. The first family contains algorithms that rely on an ansatz, such as the variational (VQE),^3−7^ contracted,^8^ and projective^9^ (PQE) quantum eigensolvers, while the second is comprised of ansatz-independent schemes, including quantum imaginary time evolution^10,11^ and quantum subspace diagonalization methods.^10,12−15^

Focusing on ansatz-dependent techniques that interest us more for the purposes of this work, the trial state is expressed in terms of a unitary parametrization, i.e.,
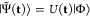
1where **t** denotes a set of parameters, and |Φ⟩ is a reference state that can be easily prepared on the quantum device, usually the Hartree–Fock Slater determinant. Chemically inspired ansätze are almost invariably based on the unitary extension^16−28^ of coupled-cluster theory^29−34^ (UCC). In general, a factorized form of the UCC unitary is adopted,
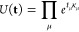
2also known as disentangled UCC,^35^ that can be readily implemented on a quantum device. The κ_μ_ symbols appearing in eq 2 represent generic Fermionic, anti-Hermitian, particle-hole excitation operators. For an *n*-tuple excitation, they are defined as
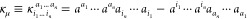
3where *a*_*p*_ (*a*^*p*^ ≡ *a*_*p*_^†^) is the second-quantized annihilation (creation) operator acting on spin orbital ϕ_*p*_, and indices *i*_1_, *i*_2_, ... or *i*, *j*, ... (*a*_1_, *a*_2_, ... or *a*, *b*, ...) label spin orbitals occupied (unoccupied) in |Φ⟩. An alternative strategy that leads to more efficient quantum circuits is to replace the Fermionic anti-Hermitian operators by their qubit counterparts, defined as
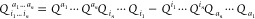
4with  denoting the qubit annihilation (creation) operator acting on the *p*^th^ qubit. However, in doing so, one may potentially sacrifice the proper sign structure of the resulting state,^36−41^ since qubit excitations neglect the Fermionic sign. Designing efficient, i.e., low-depth and noise-resilient, quantum circuits representing Fermionic and qubit excitations is crucial for the success of ansatz-dependent algorithms on current noisy quantum hardware.

Inspired by the work of Yordanov et al.,^36,37^ we have recently introduced compact Fermionic- (FEB) and qubit-excitation-based (QEB) quantum circuits that efficiently implement excitations of arbitrary many-body rank^41^ (see, also, ref (42) for an alternative CNOT-efficient approach that requires ancilla qubits). While the FEB/QEB quantum circuits are equivalent to their conventional analogs, i.e., there is no loss of accuracy in the simulations, they significantly reduce the numbers of single-qubit and, more importantly, CNOT gates (recall that experimental realizations of two-qubit gates, such as CNOT, tend to have errors that are about 10 times larger than those of the single-qubit ones^43^). For example, the standard quantum circuit implementing a hextuple qubit excitation requires more than 45,000 CNOT gates, while its QEB counterpart needs only about 2,000. Despite the drastic reduction in the CNOT count afforded by the FEB and QEB quantum circuits, their number continues to scale exponentially with the operator many-body rank. Consequently, quantum algorithms based on a full FEB/QEB operator pool will typically generate circuits with unfavorable CNOT counts when compared to approaches relying on pools containing lower-rank excitation operators, such as singles and doubles or their generalized extension.^44,45^

As elaborated on in our earlier study,^41^ the multiqubit-controlled *R*_*y*_ gate is the dominant source of CNOTs in the FEB/QEB quantum circuits. In that work, we relied on an ancilla-free implementation of that gate that requires 2^2*n*–1^ CNOTs, where *n* is the many-body rank of the given excitation operator. Adopting more efficient implementations of the multiply controlled *R*_*y*_ gate can significantly reduce the CNOT requirements. For example, the approach advocated in ref (46) results in the linear-scaling CNOT count of 12*n* – 14 but requires  ancilla qubits, where  denotes the ceiling of *x*. Recently, ancilla-free, CNOT-efficient implementations of multiply controlled gates have been proposed. Of particular interest are the methods introduced in refs (47) and (48), which decompose the multiqubit-controlled *R*_*y*_ gate into circuits with CNOT counts of 16*n*^2^ – 24*n* + 10 and, at most, 32*n* – 40, respectively. All of these state-of-the-art decompositions generate FEB/QEB quantum circuits with significantly fewer CNOT gates compared to those reported in our earlier study, especially as the many-body rank increases. Nevertheless, they either require ancilla qubits, have a  scaling, or have large prefactors in the resulting CNOT counts.

In our efforts to design CNOT-frugal FEB/QEB quantum circuits, we opted for a different strategy. In this letter, we consider approximate implementations of the multiqubit-controlled *R*_*y*_ gate in which the number of control qubits is reduced. Since the resulting circuits are not equivalent to their parent FEB/QEB counterparts, some loss of accuracy in the computed energies is anticipated. Furthermore, as shown analytically in the Supporting Information, the removal of control qubits leads to the breaking of the particle number (*N*) and total spin projection (*S*_*z*_) symmetries, while spatial symmetry is still preserved. To demonstrate this effect, we performed single-point VQE UCC with doubles (UCCD) simulations using the full QEB circuits and three approximations, the numerical results of which are depicted in Figure 1. In these illustrative calculations, we focused on the H_6_ linear chain, as described by the STO-6G minimum basis.^49^ The geometry that we selected was characterized by the distance between neighboring hydrogen atoms (*R*_H–H_) of 2.0 Å, the largest H–H separation considered in our earlier study.^41^ As shown in Figure 1, the removal of controls from the multiqubit-controlled *R*_*y*_ gate leads to a “leaking” of the wave function into other symmetry sectors of the Fock space. Specifically, we observe contaminants with eigenvalues of *N* and *S*_*z*_ that differ by ±2 and ±4 for *N*, and ±1 and ±2 au for *S*_*z*_, relative to the *N* = 6 and *S*_*z*_ = 0 au values characterizing the ground electronic state of H_6_. Furthermore, in this numerical experiment, we observe that spatial symmetry is unaffected by the removal of controls from the multiply controlled *R*_*y*_ gate. These observations are consistent with the analytical results presented in the Supporting Information. As might have been anticipated, we find that the more controls are removed, the more severe the symmetry breaking becomes, as illustrated in Figure 1.

**Figure 1:**
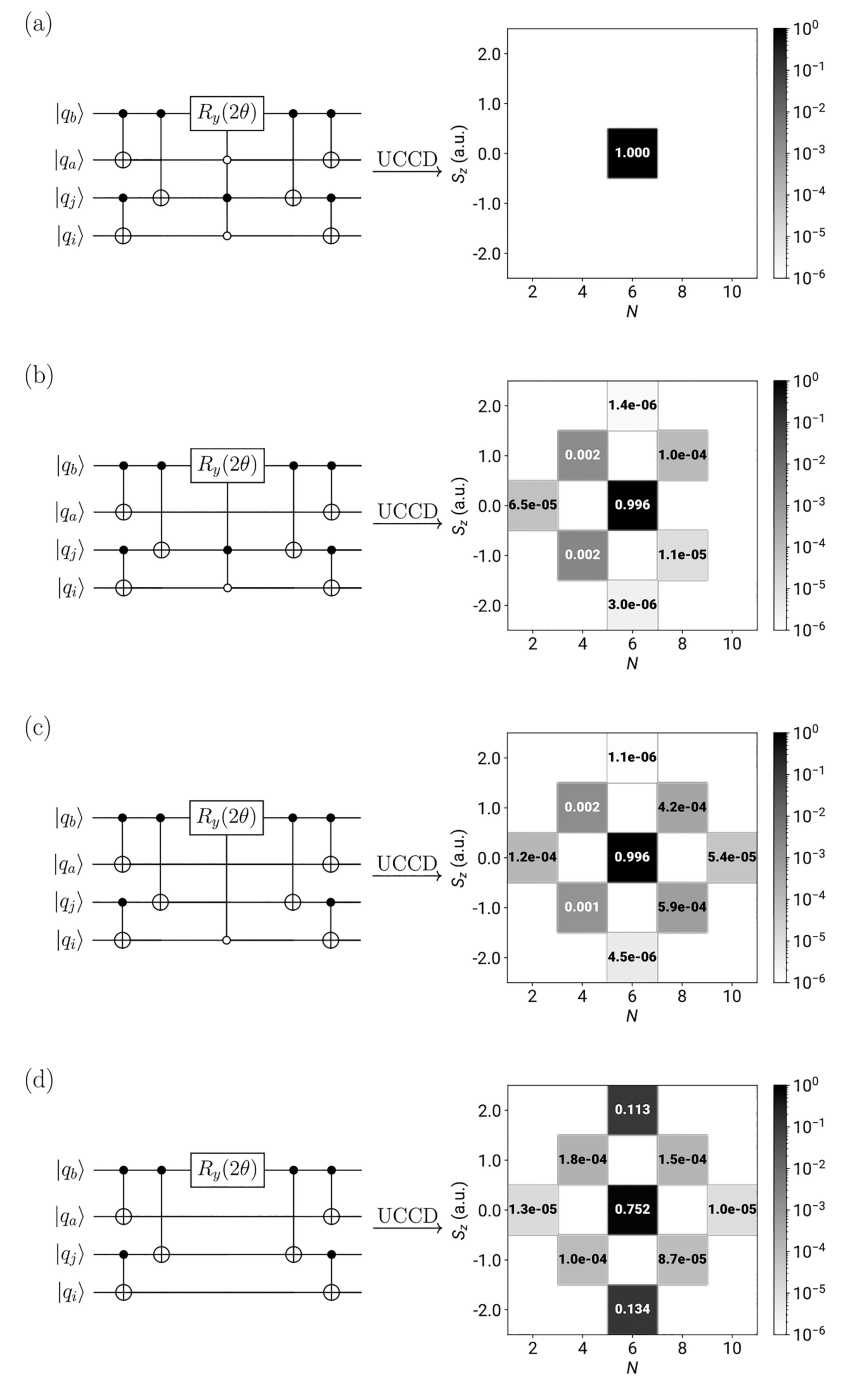
Illustration of the *N*- and *S*_*z*_-symmetry breaking introduced by the removal of (a) 0, (b) 1, (c) 2, and (d) 3 controls from the multiply controlled *R*_*y*_ gate appearing in the FEB/QEB quantum circuits implementing double excitations. On the left, we give the relevant qubit double excitation circuits, and on the right, we provide the weight of each symmetry sector of the Fock space to the converged wave functions. Since spatial symmetry is conserved, only the totally symmetric part of the Fock space is considered. The depicted data resulted from VQE QEB-UCCD simulations of the H_6_/STO-6G linear chain with a separation between neighboring H atoms of 2.0 Å.

Nevertheless, it might still be tempting to remove all controls from such multiqubit-controlled *R*_*y*_ gates and restore symmetry by, for example, postselection. As shown in the Supporting Information, such a minimalist approach not only results in significant symmetry breaking, but also generates energetics of extremely poor quality. Although restoring the *N* and *S*_*z*_ symmetries significantly improves the energy, it remains tens of millihartree away from that obtained with the full implementation.

Consequently, the guiding principle in designing such approximate FEB/QEB quantum circuits has been to find a good compromise between reducing the CNOT count and minimizing the loss of accuracy in the computed energies and breaking of symmetries in the final states. In the Supporting Information, we consider various approximate schemes, implemented in a local version of the QForte package.^50^ We performed single-point selected PQE^9^ (SPQE) simulations for the challenging H_6_/STO-6G linear chain with *R*_H–H_ = 2.0 Å. Recall that the SPQE algorithm typically relies on a complete pool of particle–hole excitation operators to iteratively construct the ansatz, eq 2, and the optimum parameters are obtained by enforcing the residual condition
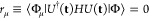
5for all excited Slater determinants |Φ_μ_⟩ corresponding to the excitation operators κ_μ_ appearing in the ansatz unitary *U*(**t**) (the details of the PQE and SPQE approaches can be found in refs (9) and (41)). Based on these preliminary computations, the best balance is offered by the following recipe (see the Supporting Information for the details):

- Single and double excitations are treated fully [see panels (a) and (b) of Figure S6].
- For triple and quadruple excitations, only controls over qubits corresponding to occupied spin orbitals are retained in the multiqubit-controlled *R*_*y*_ gate [see panels (c) and (d) of Figure S6].
- For pentuple and higher-rank excitations, all controls are removed [see Figure S6(e)], i.e., the multiqubit-controlled *R*_*y*_ gate is replaced by its single-qubit analog.

In the case of higher-rank excitation operators, the above procedure reduces the scaling of the CNOT count with the excitation rank from exponential to linear. For qubit excitations, in particular, the number of CNOT gates becomes 4*n* – 2, where *n* is the excitation rank.

To assess the effectiveness of the above approximation scheme, denoted as aFEB for Fermionic and aQEB for qubit excitations, and to compare it with the parent FEB/QEB quantum circuits across a wide range of correlation effects, we performed SPQE simulations of the symmetric dissociation of the H_6_/STO-6G linear chain. The grid of H–H distances used to sample the potential energy curve (PEC) was *R*_H–H_ = 0.5, 0.6, ..., 4.0 Å. In the Supporting Information, we also examined the dissociation process of two additional systems, both treated with an STO-6G basis. The first was the symmetric dissociation of the H_6_ ring, employing the same grid of distances between neighboring hydrogen atoms as in the case of the linear chain. The second was the *C*_2*h*_-symmetric dissociation of the H_6_ linear chain into two stretched H_3_ linear chains. To be precise, we started from the H_6_ linear chain with *R*_H–H_ = 2.0 Å, lying on the *z* axis. Subsequently, the *y* coordinate of every other H atom was gradually increased, until the final value of 3.4641016 Å. In this arrangement, the 6 H atoms form a “zig-zag” pattern composed of three equilateral triangles with sides of 4.0 Å. The *y* coordinate of the selected H atoms was uniformly sampled as  Å, with *n* = 0, 1, ..., 35. All SPQE simulations reported in this work utilized a full operator pool and micro- and macroiteration thresholds of 10^–5^*E*_*h*_ and 10^–2^*E*_*h*_, respectively (see refs (9) and (41) for the details of the recently proposed SPQE algorithm). To ensure a lower number of residual element evaluations, the PQE microiterations employed the direct inversion of the iterative subspace^51−53^ (DIIS) accelerator, and the maximum number of microiterations was set to 50. All correlated approaches were based on restricted Hartree–Fock references with the one- and two-electron integrals obtained from Psi4.^54^

We begin the discussion of our numerical results by examining the ability of the aFEB-SPQE approach to reproduce the parent FEB-SPQE simulations and to reduce the required computational resources. To that end, in Figure 2, we compare the energies, numbers of operators in the converged ansatz unitaries, CNOT counts, and numbers of residual element evaluations obtained with FEB-SPQE and aFEB-SPQE, characterizing the symmetric dissociation of the H_6_/STO-6G linear chain. A quick inspection of Figure 2 immediately reveals that aFEB-SPQE is both a highly accurate approximation of FEB-SPQE and computationally efficient. In the case of energetics, aFEB-SPQE faithfully reproduces the data of the full FEB-SPQE approach, being characterized by mean absolute, maximum absolute, and nonparallelity error values of 10, 32, and 53 μ*E*_*h*_, respectively. As far as the computational resources are concerned, aFEB-SPQE captures practically identical numbers of parameters when compared to FEB-SPQE [see panel (b) of Figure 2]. Nevertheless, as illustrated in Figure 2(c), aFEB-SPQE generates quantum circuits with significantly reduced numbers of CNOT gates than full FEB-SPQE. As might have been anticipated from the nature of the approximation, the disparity between the aFEB- and FEB-SPQE CNOT counts is dramatically increased as the strength of nondynamic correlations increases, with aFEB-SPQE requiring up to 4 times fewer CNOTs than its full FEB counterpart. Finally, the aFEB- and FEB-SPQE schemes require more or less the same numbers of residual element evaluations. Consequently, despite the drastic nature of the approximation in the quantum circuits, aFEB-SPQE accurately reproduces the FEB-SPQE energies and, by extension, those of the full configuration interaction (FCI) but at a tiny fraction of the computational cost of its FEB-SPQE parent. This observation is true for the entire range of electron correlation effects characterizing the symmetric dissociation of the H_6_/STO-6G linear chain.

**Figure 2:**
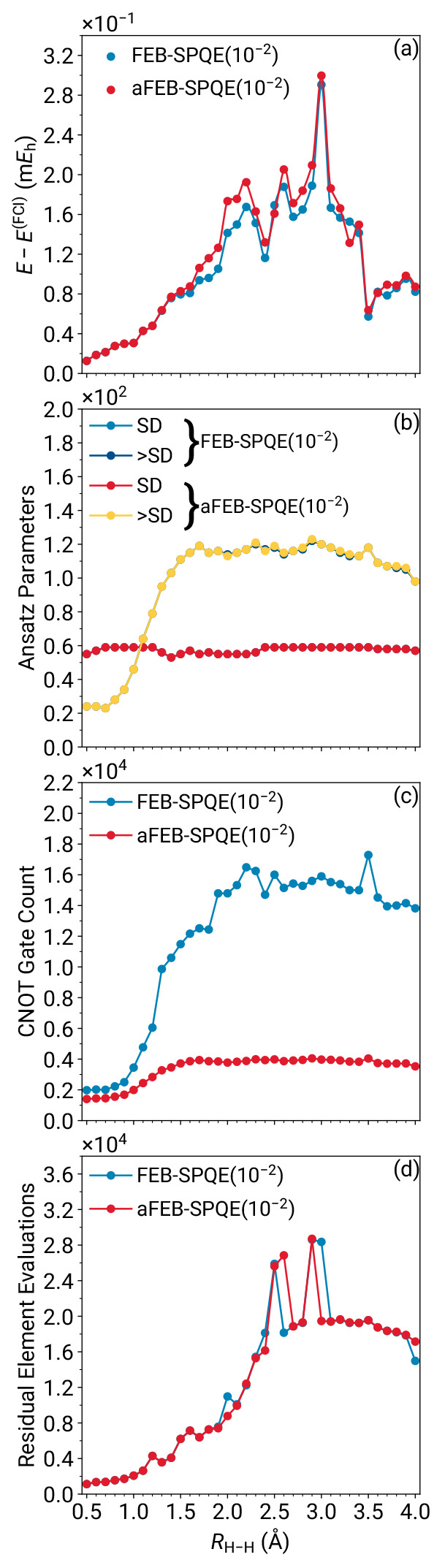
Errors relative to FCI (a), ansatz parameters (b), CNOT gate counts (c), and residual element evaluations (d) characterizing the FEB- and aFEB-SPQE simulations of the symmetric dissociation of the linear H_6_/STO-6G system. The “SD” and “>SD” symbols in the legend to panel (b) denote single or double (SD) or higher (>SD) excitation operators. Note the use of scientific notation on the *y* axis of each panel.

Despite the excellent performance in recovering the FEB-SPQE energetics, as already mentioned above and elaborated on in the Supporting Information, the approximations in the underlying quantum circuits defining the aFEB-SPQE approach result in the breaking of the particle number *N* and total spin projection *S*_*z*_ symmetries. It is thus worth examining the degree to which these symmetries are broken. As illustrated in Figure 3, the expectation values of the *N* and *S*_*z*_ operators are essentially identical to the eigenvalues of 6 and 0 au, respectively, characterizing the ground electronic state of the linear H_6_ system. Indeed, the maximum unsigned errors are 2 × 10^–5^ in the case of *N* and 3 × 10^–6^ au for *S*_*z*_. However, because the symmetry breaking introduces contaminants with both lower and higher eigenvalues of *N* and *S*_*z*_, expectation values are not a good metric. By examining the error bars shown in Figure 3, given by the standard deviation , the following trend becomes apparent. In the weakly correlated regime, there is practically no symmetry breaking. As all H–H distances are symmetrically stretched, the standard deviations gradually increase in the recoupling region until they reach their maximum values, around *R*_H–H_ = 2.5 Å. Finally, as H_6_ approaches its dissociation limit, the standard deviations gradually decrease. This pattern directly correlates with the number of higher-than-double excitation operators in the ansatz, as shown in Figure 2(b). This behavior is not surprising since the aFEB approximate scheme relies on a full implementation of singles and doubles, i.e., the higher-than-double excitation operators are the sole source of *N*- and *S*_*z*_-symmetry contaminants. The maximum standard deviations of max(σ_*N*_) = 0.011 and max(σ_*S*_*z*__) = 0.003 au are, respectively, 2 and 3 orders of magnitude smaller than the distance of 1 between the neighboring eigenvalues of *N* and *S*_*z*_. This observation provides further evidence supporting the notion that the aFEB scheme induces negligible symmetry breaking effects. As a definitive proof, we computed the weight of the totally symmetric Slater determinants with *N* = 6 and *S*_*z*_ = 0 au in the final wave functions. Focusing on the *R*_H–H_ = 2.5 and 2.8 Å geometries, corresponding to max(σ_*S*_*z*__) and max(σ_*N*_), respectively, we find that the weight of determinants having the correct symmetry properties is 99.998% and 99.999%.

**Figure 3:**
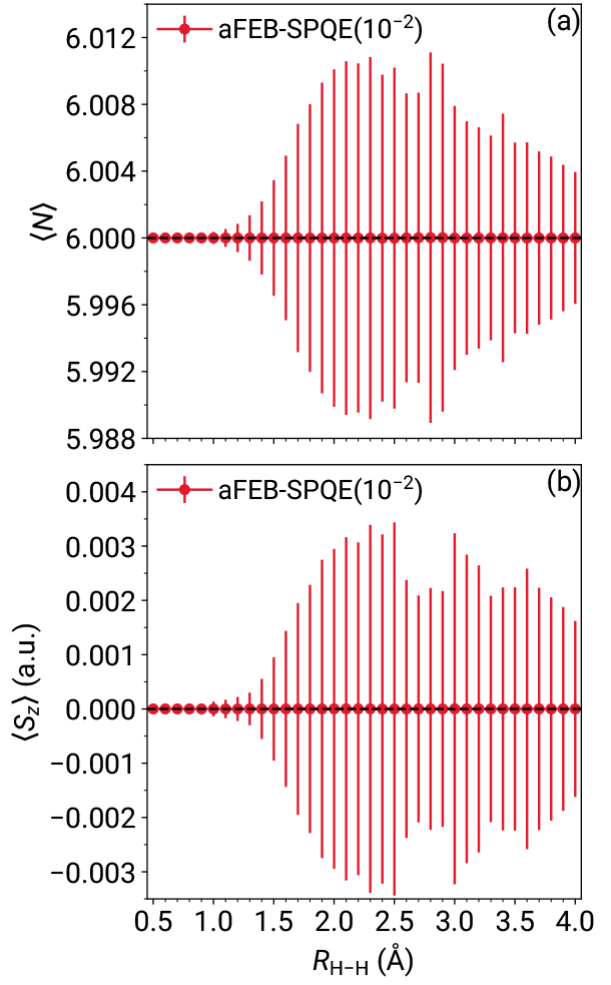
Expectation values of (a) the particle number *N* and (b) projection of the total spin on the *z* axis *S*_*z*_ operators characterizing the aFEB-SPQE simulations of the symmetric dissociation of the linear H_6_/STO-6G system. The vertical lines denote standard deviations, computed as . The horizontal dashed lines denote the corresponding eigenvalues for the ground electronic state of the H_6_/STO-6G linear chain.

Due to the use of a determinantal basis, the converged states resulting from FEB- and aFEB-SPQE simulations are not necessarily eigenfunctions of the square of the total spin operator, *S*^2^. Nevertheless, it is still interesting to examine how the ⟨*S*^2^⟩ and σ_*S*^2^_ values are affected when one transitions from the parent FEB-SPQE scheme to the aFEB approximation. As depicted in Figure 4, aFEB-SPQE yields nearly identical ⟨*S*^2^⟩ and σ_*S*^2^_ values to those obtained with the full FEB-SPQE approach. This further reinforces the fact that aFEB-SPQE is a high-fidelity approximation to FEB-SPQE.

**Figure 4:**
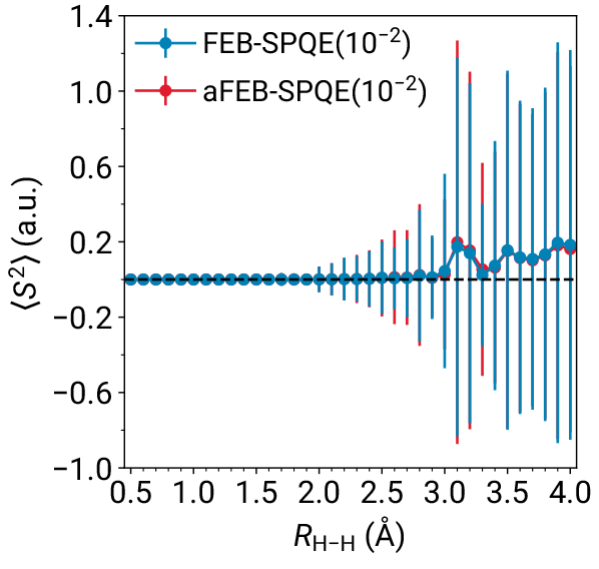
Expectation values of the total spin squared *S*^2^ operator characterizing the FEB- and aFEB-SPQE simulations of the symmetric dissociation of the linear H_6_/STO-6G system. The vertical lines denote standard deviations, computed as . The horizontal dashed line denotes the corresponding eigenvalue for the ground electronic state of the H_6_/STO-6G linear chain.

Although here we focused on the aFEB-/FEB-SPQE pair, as shown in Figures S7–S9, similar observations can be made when examining the performance of the aQEB approximation to QEB-SPQE. In comparing the two approximate schemes among themselves (Figures S10 and S11), we notice that aQEB-SPQE typically produces quantum circuits with fewer CNOT gates than its Fermionic counterpart, especially in situations characterized by stronger nondynamic correlation effects. At the same time, however, aQEB-SPQE is typically less accurate than aFEB-SPQE, and the symmetry breaking is more pronounced. These observations indicate that aFEB-SPQE achieves a favorable balance between minimizing the CNOT count and mitigating the loss of accuracy in energetics and symmetry breaking in the final states.

In the Supporting Information, we also examined the performance of aFEB-SPQE on the symmetric dissociation of the H_6_ ring and the *C*_2*h*_-symmetric dissociation of the “zig-zag” H_6_ system, both treated with an STO-6G basis. These hydrogen clusters and their linear chain isomer serve as prototypical systems for strong correlations. A quick inspection of Figures S12–S17 immediately reveals that aFEB-SPQE performs equally well on these two challenging systems. In particular, aFEB-SPQE faithfully reproduces the parent FEB-SPQE energies while requiring up to 4 times fewer CNOT gates. Furthermore, the symmetry-breaking introduced by the aFEB-SPQE approximation is essentially negligible, rendering symmetry restoration arguments unnecessary. These observations point toward the stability of the aFEB approximation to FEB, although further investigation is needed.

Our preliminary numerical results advocate that the aFEB scheme has several desirable properties of an approximation. It is highly accurate, reproducing the parent FEB-SPQE simulations with errors not exceeding a few microhartree. It has a low computational cost, reducing the number of CNOT gates compared to its already efficient FEB-SPQE analog by at most 75% (65% on average). Furthermore, the aFEB quantum circuits are much simpler compared to their FEB counterparts, suggesting an easier hardware implementation. One aspect of aFEB-SPQE that we intend to examine in the future is its stability. Although preliminary single-point calculations for the H_8_ linear chain, the linear BeH_2_ system, and the *C*_2*v*_-symmetric insertion of Be to H_2_ indicate that aFEB-SPQE behaves similarly to the case of the H_6_ linear chain, a more thorough investigation is required. It is also worth exploring the usefulness of symmetry restoration^55−57^ within the various approximations considered in this work. As shown in our preliminary single-point calculations reported in Table S1, restoring the *N* and *S*_*z*_ symmetries in aFEB-/aQEB-SPQE has a negligible effect on the computed energies. This is due to the fact that the symmetry breaking in these approximations is practically insignificant. Nevertheless, symmetry restoration might prove useful in the context of more drastic approximations. In such cases, it might be possible to reduce the CNOT counts even further while still maintaining a high degree of accuracy in the computed energies.

## Supporting Information Available

Details on the approximate Fermionic- and qubit-excitation-based quantum circuits, analysis of the ensuing symmetry breaking, results of additional numerical simulations (PDF)
Numerical data generated in this study (XLSX)
