# Supplementary material for: Linear-Scaling Quantum Circuits for Computational Chemistry
Source: J Chem Theory Comput. 2023 Jul 6;19(15):4815–21. doi: 10.1021/acs.jctc.3c00376 (PMC10413858; doi:10.1021/acs.jctc.3c00376)
Supplement: Supplementary file 1 — ct3c00376_si_001.pdf [file ct3c00376_si_001.pdf]

# Supporting Information: Linear-Scaling Quantum Circuits for Computational Chemistry

Ilias Magoulas* and Francesco A. Evangelista*

*Department of Chemistry and Cherry Emerson Center for Scientific Computation, Emory University, Atlanta, Georgia 30322, USA*

E-mail: ilias.magoulas@emory.edu; francesco.evangelista@emory.edu

This Supporting Information document is organized as follows. In Section S1 we describe the various approximate fermionic- (FEB) and qubit-excitation-based (QEB) quantum circuits considered in this study, including an analysis of the ensuing symmetry breaking. Section S2 provides, in a graphical form, the results of our additional numerical simulations. In particular, in Section S2.1 we assess the effectiveness of the aQEB approximation to QEB-SPQE($10^{-2}$) for the symmetric dissociation of the $H_6$/STO-6G linear chain. In Section S2.2, we compare the performance of the aFEB- and aQEB-SPQE($10^{-2}$) approximations for the symmetric dissociation of the $H_6$/STO-6G linear chain. Finally, in Sections S2.3 and S2.4 we examine the performance of the aFEB approximation to FEB-SPQE($10^{-2}$) for the dissociation of the ring and "zig-zag" $H_6$/STO-6G isomers, respectively.

The numerical data generated in this study can be found in the Excel file that forms part of the present Supporting Information.

# S1 Approximate FEB/QEB Quantum Circuits

In this section, we discuss the various approximate FEB/QEB quantum circuits explored in this study. Figure S1 depicts the full QEB [panel (a)] and FEB [panel (b)] quantum circuits performing $n$-tuple qubit and fermionic excitations, respectively. As shown in ref 41 of the main text, the decomposition of the multi-qubit-controlled $R_y$ gate introduces an exponential number of CNOT gates, rendering it the main source of CNOTs. This is still true even if one adopts the more efficient implementations of the multiply controlled $R_y$ gate introduced in refs 46–48 of the main text. The number of CNOTs can be dramatically reduced by adopting approximate forms of the multi-qubit-controlled $R_y$ gate in which certain controls are removed.

Without loss of generality, to illustrate the consequences of approximate implementations of the multi-qubit-controlled $R_y$ gate, we focus on the simpler case of QEB doubles. The full

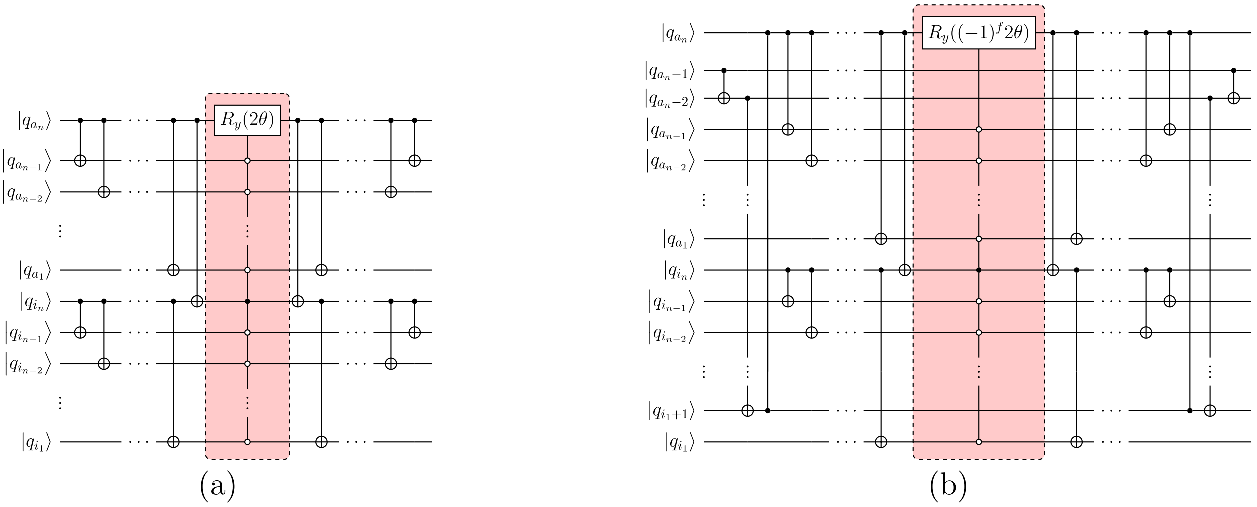


Figure S1: CNOT-efficient quantum circuits performing (a) qubit and (b) fermionic $n$-tuple particle–hole excitations. Indices $i_1, \ldots, i_n$ $(a_1, \ldots, a_n)$ correspond to spinorbitals occupied (unoccupied) in the reference Slater determinant. The parameter $f$ controlling the sign of the rotation angle depends on the excitation rank $n$ as follows: $f = 0$ for $n = 1, 4, 5, 8, 9, \ldots$ and $f = 1$ for $n = 2, 3, 6, 7, \ldots$. In both circuits, the multi-qubit-controlled $R_y$ gate, shaded in red color, is the main source of CNOT gates. The open circles denote anticontrol qubits.

circuit, shown in Figure S2, implements a double qubit excitation exactly, i.e.,

$$e^{Q_{ij}^{ab}} |q_i q_j q_a q_b\rangle = \begin{cases} \cos(\theta)\,|1_i 1_j 0_a 0_b\rangle + \sin(\theta)\,|0_i 0_j 1_a 1_b\rangle, & q_i = q_j = 1, q_a = q_b = 0 \\ -\sin(\theta)\,|1_i 1_j 0_a 0_b\rangle + \cos(\theta)\,|0_i 0_j 1_a 1_b\rangle, & q_i = q_j = 0, q_a = q_b = 1 \\ |q_i q_j q_a q_b\rangle, & \text{otherwise} \end{cases} \cdot \quad \text{(S1)}$$

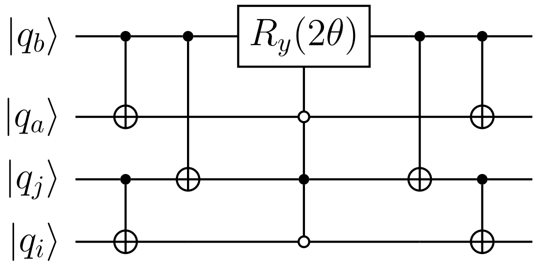


Figure S2: QEB quantum circuit performing a qubit double particle–hole excitation, $\exp(Q_{ij}^{ab})$.

Essentially, the QEB doubles circuit performs a continuous exchange of the $|1_i 1_j 0_a 0_b\rangle$ and

$|0_i 0_j 1_a 1_b\rangle$ states. Figure S3 depicts the crudest approximation in which all controls have been removed, replacing the multi-qubit-controlled $R_y$ gate by its single-qubit counterpart.

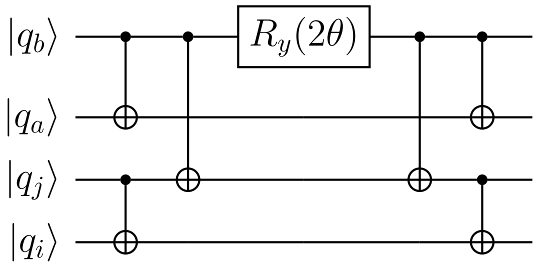


Figure S3: In the crudest approximation to the QEB quantum circuit shown in Figure S2, the multi-qubit-controlled $R_y$ gate is replaced by its single-qubit counterpart.

It is straightforward to show that this quantum circuit performs the operation

$$U\left|q_iq_jq_aq_b\right\rangle = \begin{cases} \cos(\theta)\left|0_i0_j0_a0_b\right\rangle + \sin(\theta)\left|1_i1_j1_a1_b\right\rangle, & q_i=q_j=q_a=q_b=0 \\ -\sin(\theta)\left|0_i0_j0_a0_b\right\rangle + \cos(\theta)\left|1_i1_j1_a1_b\right\rangle, & q_i=q_j=q_a=q_b=1 \\ \cos(\theta)\left|1_i0_j0_a0_b\right\rangle + \sin(\theta)\left|0_i1_j1_a1_b\right\rangle, & q_i=1, q_j=q_a=q_b=0 \\ -\sin(\theta)\left|1_i0_j0_a0_b\right\rangle + \cos(\theta)\left|0_i1_j1_a1_b\right\rangle, & q_i=0, q_j=q_a=q_b=1 \\ \cos(\theta)\left|0_i1_j0_a0_b\right\rangle + \sin(\theta)\left|1_i0_j1_a1_b\right\rangle, & q_j=1, q_i=q_a=q_b=0 \\ -\sin(\theta)\left|0_i1_j0_a0_b\right\rangle + \cos(\theta)\left|1_i0_j1_a1_b\right\rangle, & q_j=0, q_i=q_a=q_b=1 \\ \cos(\theta)\left|1_i1_j0_a0_b\right\rangle + \sin(\theta)\left|0_i0_j1_a1_b\right\rangle, & q_i=q_j=1, q_a=q_b=0 \\ -\sin(\theta)\left|1_i1_j0_a0_b\right\rangle + \cos(\theta)\left|0_i0_j1_a1_b\right\rangle, & q_i=q_j=0, q_a=q_b=1 \\ \cos(\theta)\left|0_i0_j1_a0_b\right\rangle + \sin(\theta)\left|1_i1_j0_a1_b\right\rangle, & q_a=1, q_i=q_j=q_b=0 \\ -\sin(\theta)\left|0_i0_j1_a0_b\right\rangle + \cos(\theta)\left|1_i1_j0_a1_b\right\rangle, & q_a=0, q_i=q_j=q_b=1 \\ \cos(\theta)\left|1_i0_j1_a0_b\right\rangle + \sin(\theta)\left|0_i1_j0_a1_b\right\rangle, & q_i=q_a=1, q_j=q_b=0 \\ -\sin(\theta)\left|1_i0_j1_a0_b\right\rangle + \cos(\theta)\left|0_i1_j0_a1_b\right\rangle, & q_i=q_a=0, q_j=q_b=1 \\ \cos(\theta)\left|0_i1_j1_a0_b\right\rangle + \sin(\theta)\left|1_i0_j0_a1_b\right\rangle, & q_i=q_b=0, q_j=q_a=1 \\ -\sin(\theta)\left|0_i1_j1_a0_b\right\rangle + \cos(\theta)\left|1_i0_j0_a1_b\right\rangle, & q_i=q_b=1, q_j=q_a=0 \\ \cos(\theta)\left|1_i1_j1_a0_b\right\rangle + \sin(\theta)\left|0_i0_j0_a1_b\right\rangle, & q_i=q_j=q_a=1, q_b=0 \\ -\sin(\theta)\left|1_i1_j1_a0_b\right\rangle + \cos(\theta)\left|0_i0_j0_a1_b\right\rangle, & q_i=q_j=q_a=0, q_b=1 \end{cases}. \tag{S2}$$

The unitary operator $U$ that performs the above continuous exchanges is

$$U = \underbrace{\overbrace{e^{\theta Q^{ijab}}}^{N\pm 4}\,\overbrace{e^{\theta Q_i^{jab}}\,e^{\theta Q_j^{iab}}\,e^{\theta Q_a^{ijb}}\,e^{\theta Q_{ija}^{b}}}^{N\pm 2}\,e^{\theta Q_{ia}^{jb}}e^{\theta Q_{ja}^{ib}}}_{\text{May break } S_z \text{ symmetry: } M_S, M_S\pm 1, M_s\pm 2}\,e^{\theta Q_{ij}^{ab}}. \tag{S3}$$

(In the original, the overbrace labelled $N$ spans $e^{\theta Q_{ia}^{jb}}e^{\theta Q_{ja}^{ib}}e^{\theta Q_{ij}^{ab}}$.)

Examination of eq (S3) reveals that $U$ is the product of eight exponentials. Of these, five

violate the particle number ($N$) symmetry, attaching/ionizing either 2 or 4 electrons. At the same time, the first seven exponentials in eq (S3) have the potential to break the total spin projected on the z axis ($S_z$) symmetry, introducing contaminants with $M_S \pm 1$ and $M_S \pm 2$. Which of the seven exponentials introduce $S_z$-symmetry contaminants depends on the $m_s$ values of the spinorbitals involved in a given excitation process. Note, however, that spatial symmetry is retained.

In searching for an approximation scheme, it is crucial to not only minimize the CNOT count, but also keep the loss of accuracy in the computed energies and breaking of symmetry in the final states to a minimum. In Table S1, we considered 8 such approaches and applied them to the $H_6$/STO-6G linear chain, a prototypical strongly correlated system. In this preliminary numerical exploration, we focused on the geometry in which the separation between neighboring H atoms is $R_{\text{H–H}} = 2.0$ Å, the largest distance considered in our earlier study, ref 41 of the main text. A simple inspection of Table S1 reveals that approximation schemes with a full treatment of singles and doubles reproduce the results of the parent FEB- and QEB-SPQE methods within a fraction of a millihartree. At the same time, the symmetry breaking introduced by these approaches is practically negligible, as evidenced by the standard deviations of $N$ and $S_z$, as well as the contributions of the various symmetry sectors of the Fock space in the converged wavefunctions, shown in Figures S4 and S5. The necessity of treating single and double excitations fully, in particular the latter, is not a coincidence. Based on single-point FEB-SPQE calculations of other systems characterized by significant non-dynamic correlations, including the $H_6$ ring, the $H_8$ linear chain, the linear $BeH_2$ system, and the $C_{2\text{v}}$-symmetric insertion of Be to $H_2$, the largest double-excitation amplitudes were about 10 times greater than their higher-rank counterparts. Out of the five approximation schemes that worked well, the one that we selected to test further in the main text is defined in Figure S6. Based on the preliminary data collected in Table S1, this scheme, abbreviated as aFEB and aQEB for fermionic and qubit excitations, respectively, offers the best balance between minimizing the CNOT count and mitigating the loss of accuracy in

energetics and symmetry breaking in the final states.

In Table S1, we also examined the effect of restoring the $N$ and $S_z$ symmetries on the resulting energetics. For the more robust approximations, where singles and doubles are treated fully, symmetry restoration has a negligible impact on the energies. However, when considering the more drastic approximations in Table S1, restoring the $N$ and $S_z$ symmetries does improve the quality of the energies, especially for the minimalist scheme where all controls are removed. Nevertheless, relying solely on symmetry restoration is insufficient to obtain meaningful results, as indicated by the significant deviations observed relative to the parent data (see Table S1). Therefore, it is crucial to seek approximation schemes that not only reduce the CNOT counts but also yield highly accurate energetics with minimal symmetry breaking.

Table S1: Total electronic energies (in $E_{\mathrm{h}}$), expectation values $\langle A \rangle$ and standard deviations $\sigma_A$ of the particle number $N$, z-component of total spin $S_z$, and total spin squared $S^2$ operators (in a.u.), CNOT counts, and numbers of parameters characterizing the various full and approximate SPQE($10^{-2}$) computations of the linear $H_6$/STO-6G chain with $R_{\mathrm{H–H}} = 2.0$ Å.

| Scheme | $E$ | $E_{\mathrm{pure}}{}^{a}$ | $\langle N \rangle$ | $\sigma_N$ | $\langle S_z \rangle$ | $\sigma_{S_z}$ | $\langle S^2 \rangle$ | $\sigma_{S^2}$ | CNOTs | Parameters |
|---|---|---|---|---|---|---|---|---|---|---|
| | | | | | FEB | | | | | |
| full[b] | -2.873932 | -2.873932 | 6.000000 | 0.000000 | 0.000000 | 0.000000 | 0.000788 | 0.067510 | 14794 | 169 |
| SD-full+occ[c] | -2.873908 | -2.873931 | 5.999994 | 0.010081 | 0.000001 | 0.002952 | 0.000830 | 0.068874 | 4034 | 169 |
| SD-full+TQ-occ[d] | -2.873900 | -2.873922 | 5.999994 | 0.010099 | 0.000001 | 0.002946 | 0.000816 | 0.067949 | 3774 | 168 |
| SD-full+unocc[e] | -2.873885 | -2.873921 | 5.999994 | 0.013759 | 0.000001 | 0.002696 | 0.000794 | 0.067088 | 3296 | 168 |
| SD-full+TQ-unocc[f] | -2.873884 | -2.873921 | 5.999994 | 0.013721 | 0.000001 | 0.002690 | 0.000794 | 0.067093 | 3184 | 168 |
| SD-full[g] | -2.873695 | -2.873917 | 6.000013 | 0.027742 | -0.000051 | 0.016882 | 0.001277 | 0.081575 | 2582 | 168 |
| occ[h] | -2.861721 | -2.867900 | 5.995097 | 0.139851 | -0.001202 | 0.068937 | 0.383478 | 1.457258 | 3778 | 167 |
| unocc[i] | -2.857341 | -2.862585 | 6.002553 | 0.132236 | -0.001621 | 0.060996 | 0.539628 | 1.706678 | 2810 | 162 |
| min[j] | -2.787782 | -2.836626 | 6.005907 | 0.249094 | 0.116015 | 0.882393 | 1.283279 | 2.437863 | 2094 | 161 |
| | | | | | QEB | | | | | |
| full[b] | -2.873933 | -2.873933 | 6.000000 | 0.000000 | 0.000000 | 0.000000 | 0.002119 | 0.076628 | 14988 | 178 |
| SD-full+occ[c] | -2.873904 | -2.873949 | 6.000035 | 0.015015 | 0.000009 | 0.004386 | 0.001892 | 0.068447 | 3726 | 179 |
| SD-full+TQ-occ[d] | -2.873895 | -2.873944 | 6.000036 | 0.015420 | 0.000008 | 0.004631 | 0.001747 | 0.071800 | 3502 | 179 |
| SD-full+unocc[e] | -2.873863 | -2.873929 | 5.999952 | 0.016943 | -0.000002 | 0.005175 | 0.001884 | 0.071776 | 2948 | 178 |
| SD-full+TQ-unocc[f] | -2.873859 | -2.873927 | 5.999953 | 0.017116 | -0.000001 | 0.005307 | 0.001889 | 0.072632 | 2836 | 178 |
| SD-full[g] | -2.873390 | -2.873824 | 5.999976 | 0.037654 | -0.000049 | 0.021261 | 0.002157 | 0.091124 | 2180 | 178 |
| occ[h] | -2.864504 | -2.868381 | 5.996943 | 0.112104 | -0.000072 | 0.054616 | 0.336046 | 1.370817 | 3388 | 170 |
| unocc[i] | -2.860408 | -2.861957 | 6.000399 | 0.076798 | 0.000054 | 0.034511 | 0.684714 | 1.903075 | 2202 | 152 |
| min[j] | -2.784765 | -2.830128 | 5.996454 | 0.193159 | 0.113565 | 0.916424 | 1.468346 | 2.564975 | 1578 | 161 |

[a] These energies were obtained as the expectation value of the Hamiltonian with respect to the state in which the $N$- and $S_z$-symmetry contaminants are removed and the resulting wavefunction is normalized. [b] Full FEB/QEB circuits; no approximations. [c] Full FEB/QEB circuits for singles and doubles; for higher-than-double excitations only controls over occupied spinorbitals are retained. [d] Full FEB/QEB circuits for singles and doubles, for triples and quadruples only controls over occupied spinorbitals are retained; for pentuples and higher-rank excitations all controls are removed. [e] Full FEB/QEB circuits for singles and doubles; for higher-than-double excitations only controls over unoccupied spinorbitals are retained. [f] Full FEB/QEB circuits for singles and doubles, for triples and quadruples only controls over unoccupied spinorbitals are retained; for pentuples and higher-rank excitations all controls are removed. [g] Full FEB/QEB circuits for singles and doubles; for higher-than-double excitations all controls are removed. [h] Only controls over occupied spinorbitals are retained. [i] Only controls over unoccupied spinorbitals are retained. [j] All controls are removed.

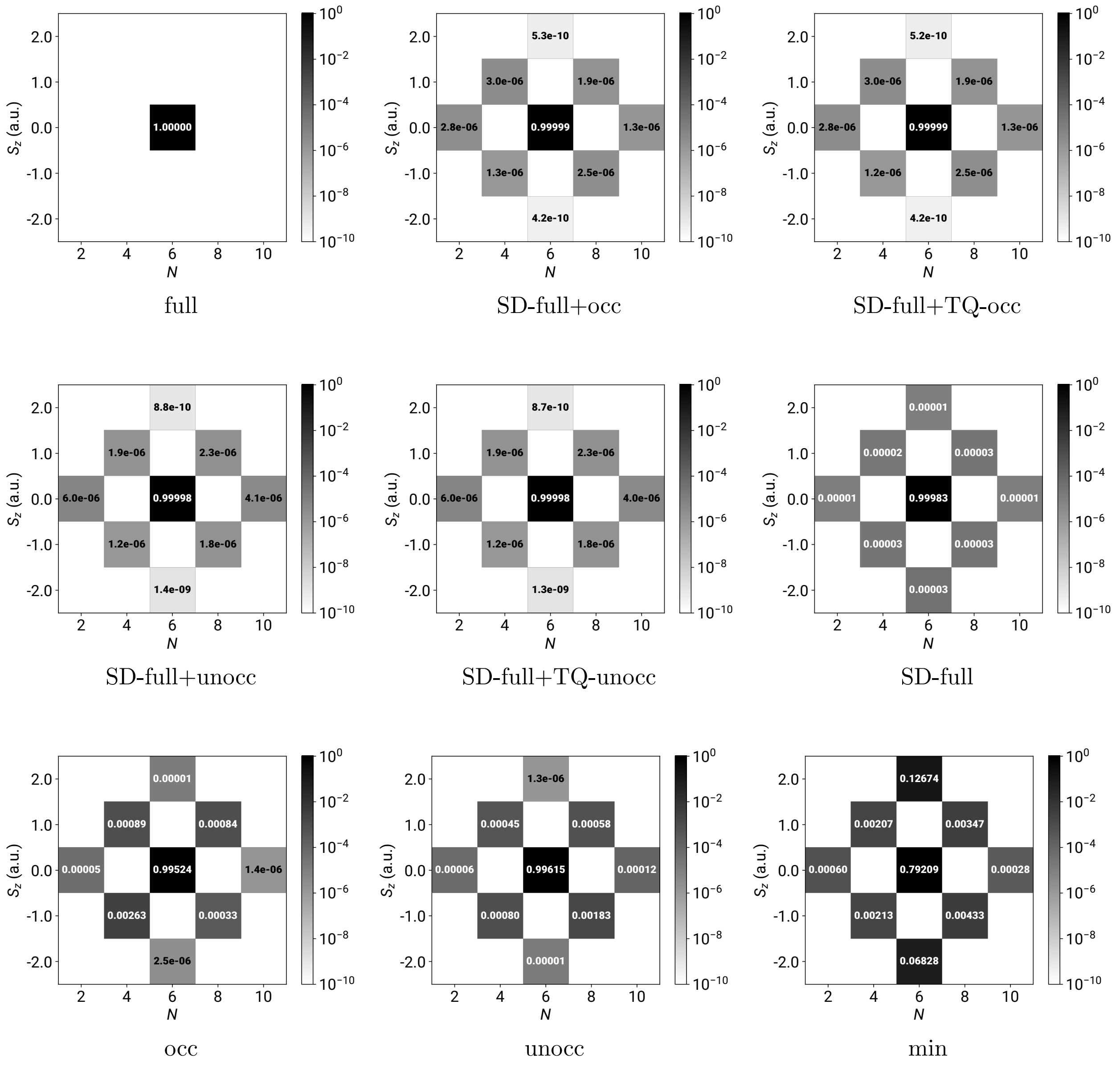


Figure S4: Contributions of the various symmetry sectors of the Fock space to the converged wavefunctions characterizing the various full and approximate FEB-SPQE($10^{-2}$) computations of the linear $H_6$/STO-6G chain with $R_{\mathrm{H-H}} = 2.0$ Å. The definitions of the various schemes can be found in the footnotes to Table S1. Note that all depicted symmetry sectors are totally symmetric since spatial symmetry is preserved.

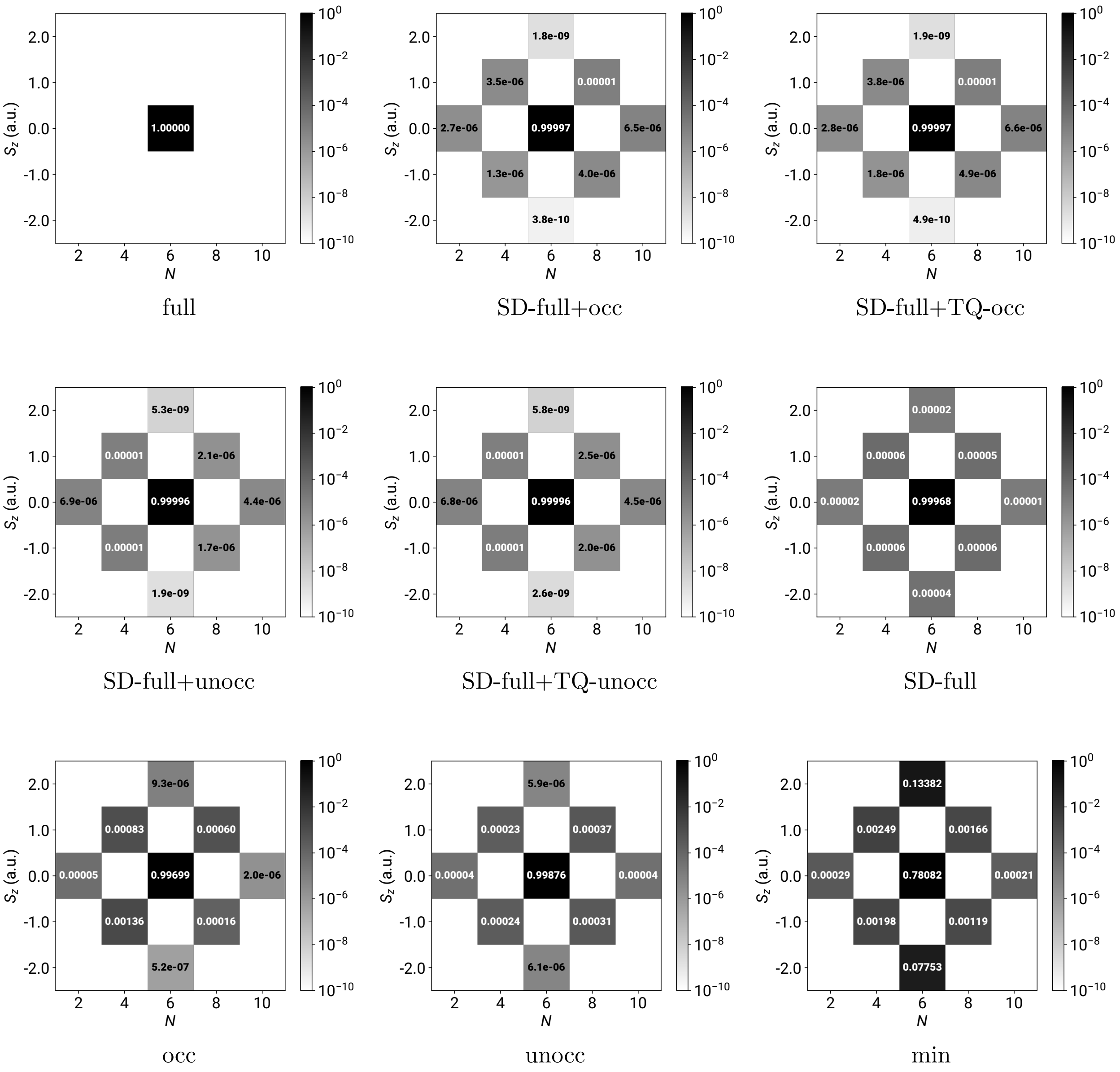


Figure S5: Contributions of the various symmetry sectors of the Fock space to the converged wavefunctions characterizing the various full and approximate QEB-SPQE($10^{-2}$) computations of the linear $H_6$/STO-6G chain with $R_{\text{H–H}} = 2.0\,\text{Å}$. The definitions of the various schemes can be found in the footnotes to Table S1. Note that all depicted symmetry sectors are totally symmetric since spatial symmetry is preserved.

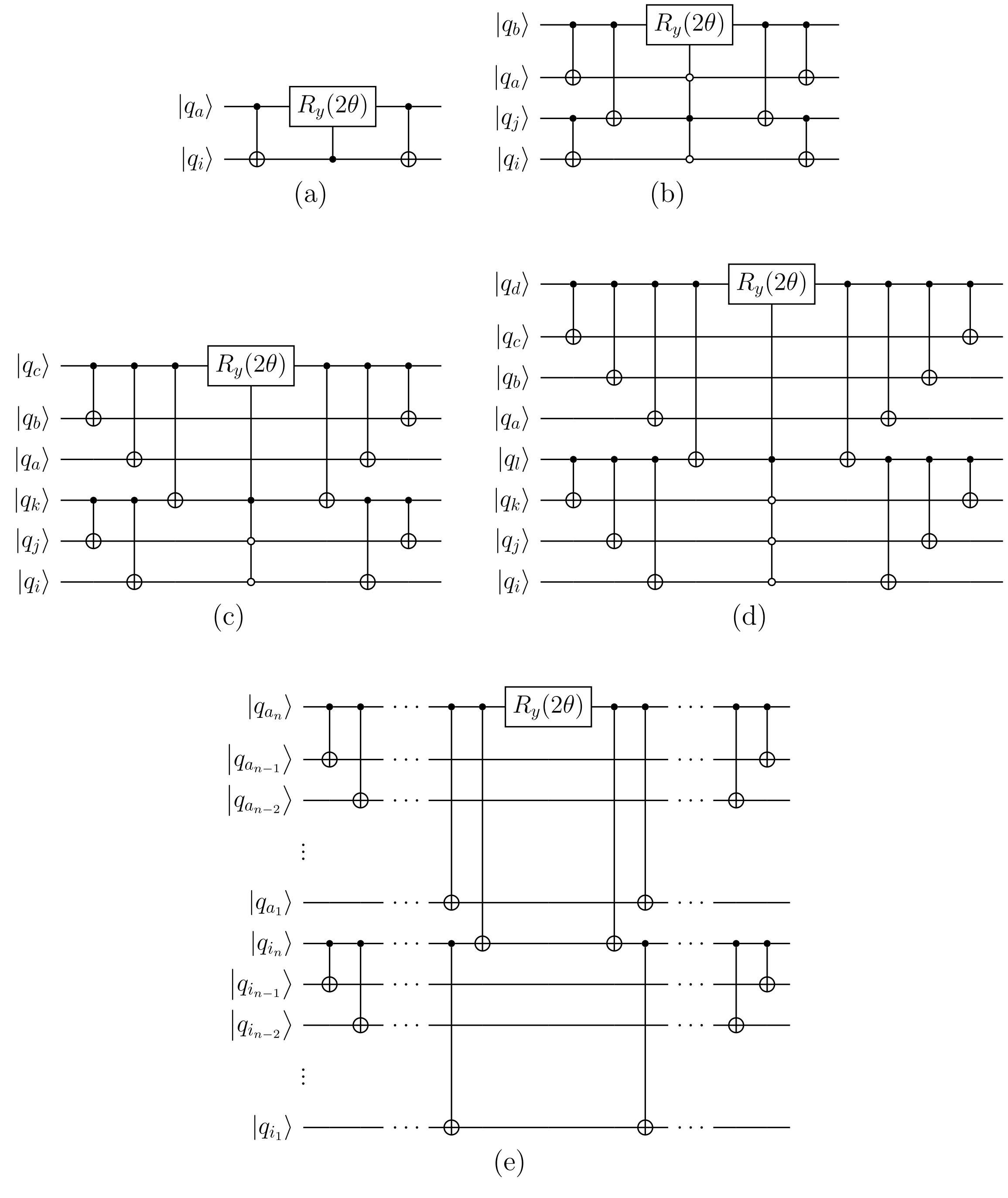


Figure S6: Quantum circuits defining the aQEB scheme. Single (a) and double (b) excitations are treated fully. For triple (c) and quadruple (d) excitations, only controls over qubits corresponding to occupied orbitals are retained in the multi-qubit-controlled $R_y$ gate. For pentuple and higher-rank excitations (e), all controls are removed, i.e., the multi-qubit-controlled $R_y$ gate is replaced by its single-qubit counterpart. The aFEB scheme is defined in a similar manner.

# S2 Results of Additional Numerical Simulations

## S2.1 Linear $H_6$/STO-6G: QEB-SPQE vs aQEB-SPQE

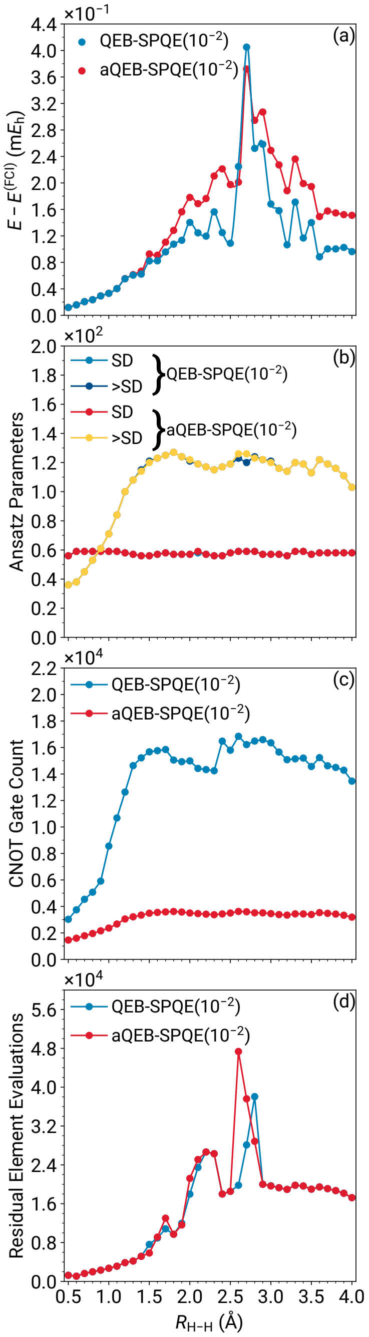


Figure S7: Errors relative to FCI [(a)], ansatz parameters [(b)], CNOT gate counts [(c)], and residual element evaluations [(d)] characterizing the QEB- and aQEB-SPQE($10^{-2}$) simulations of the symmetric dissociation of the linear $H_6$/STO-6G system. The "SD" and ">SD" symbols denote single or double (SD) or higher (>SD) excitation operators.

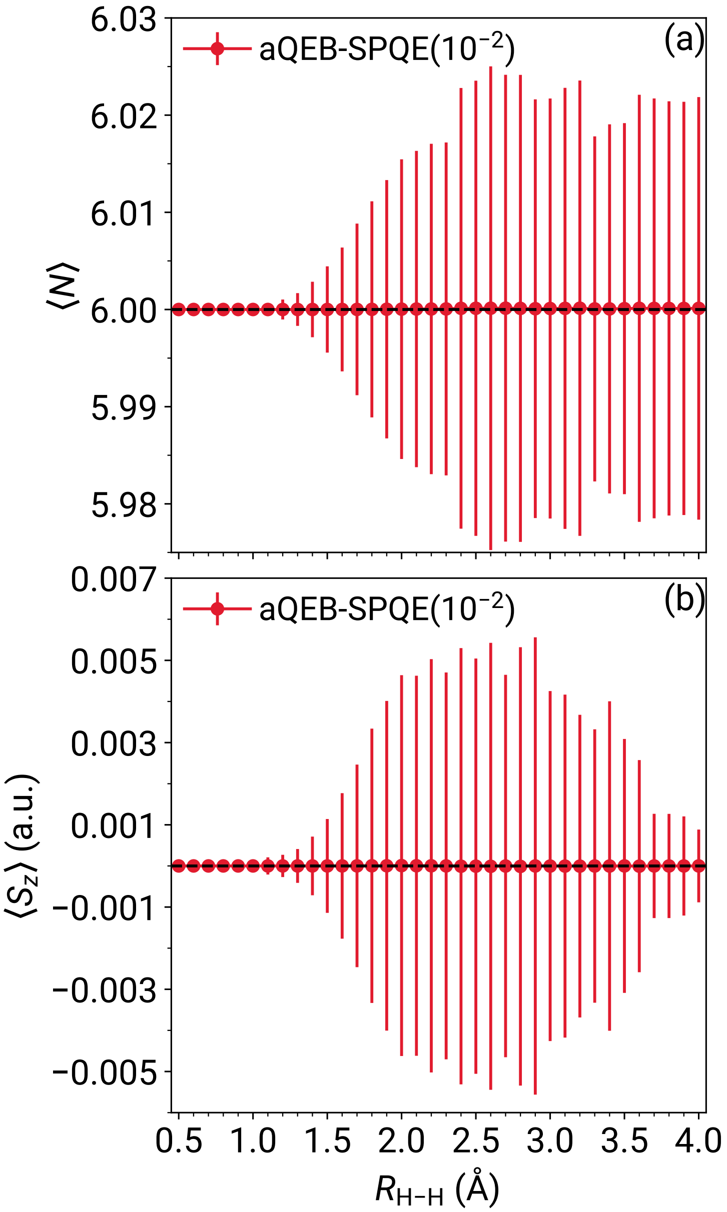


Figure S8: Expectation values of (a) the particle number $N$ and (b) the projection of the total spin on the $z$ axis $S_z$ operators characterizing the aQEB-SPQE($10^{-2}$) simulations of the symmetric dissociation of the linear $H_6$/STO-6G system. The vertical lines denote standard deviations, computed as $\sigma_A = \sqrt{\langle A^2 \rangle - \langle A \rangle^2}$. The horizontal dashed lines denote the corresponding eigenvalues for the ground electronic state of the $H_6$/STO-6G linear chain.

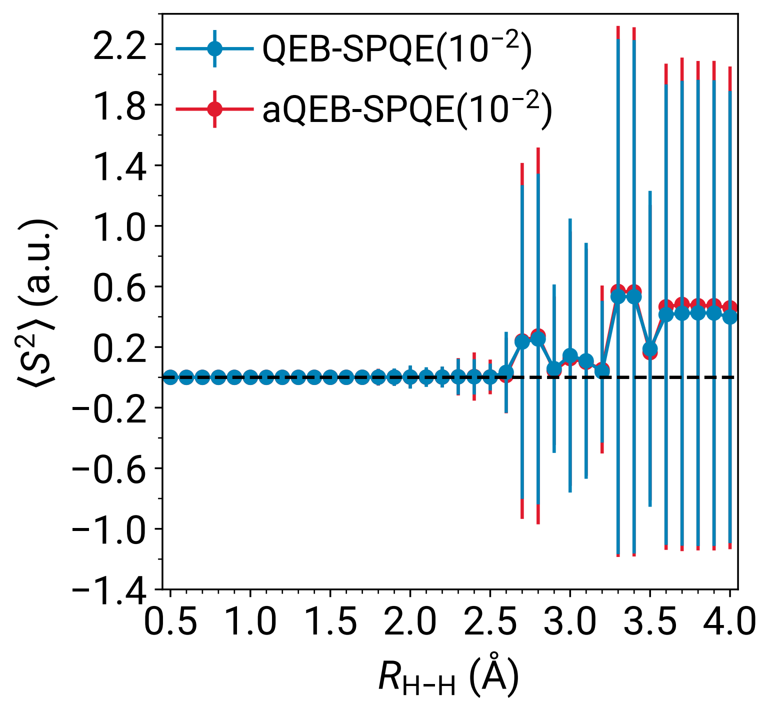


Figure S9: Expectation values of the total spin squared $S^2$ operator characterizing the QEB- and aQEB-SPQE($10^{-2}$) simulations of the symmetric dissociation of the linear $H_6$/STO-6G system. The vertical lines denote standard deviations, computed as $\sigma_A = \sqrt{\langle A^2 \rangle - \langle A \rangle^2}$. The horizontal dashed line denotes the corresponding eigenvalue for the ground electronic state of the $H_6$/STO-6G linear chain.

## S2.2 Linear $H_6$/STO-6G: aFEB-SPQE vs aQEB-SPQE

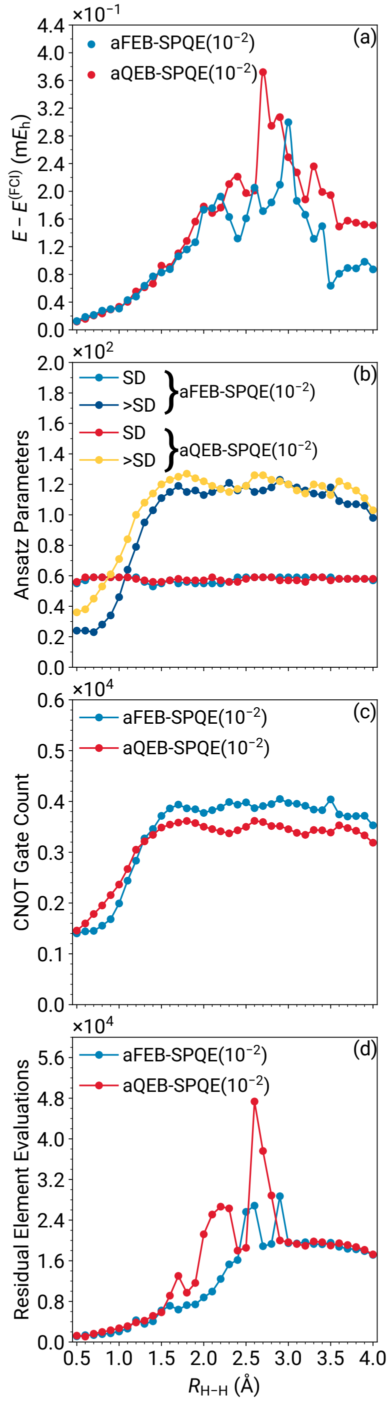


Figure S10: Errors relative to FCI [(a)], ansatz parameters [(b)], CNOT gate counts [(c)], and residual element evaluations [(d)] characterizing the aFEB- and aQEB-SPQE($10^{-2}$) simulations of the symmetric dissociation of the linear $H_6$/STO-6G system. The "SD" and ">SD" symbols denote single or double (SD) or higher (>SD) excitation operators.

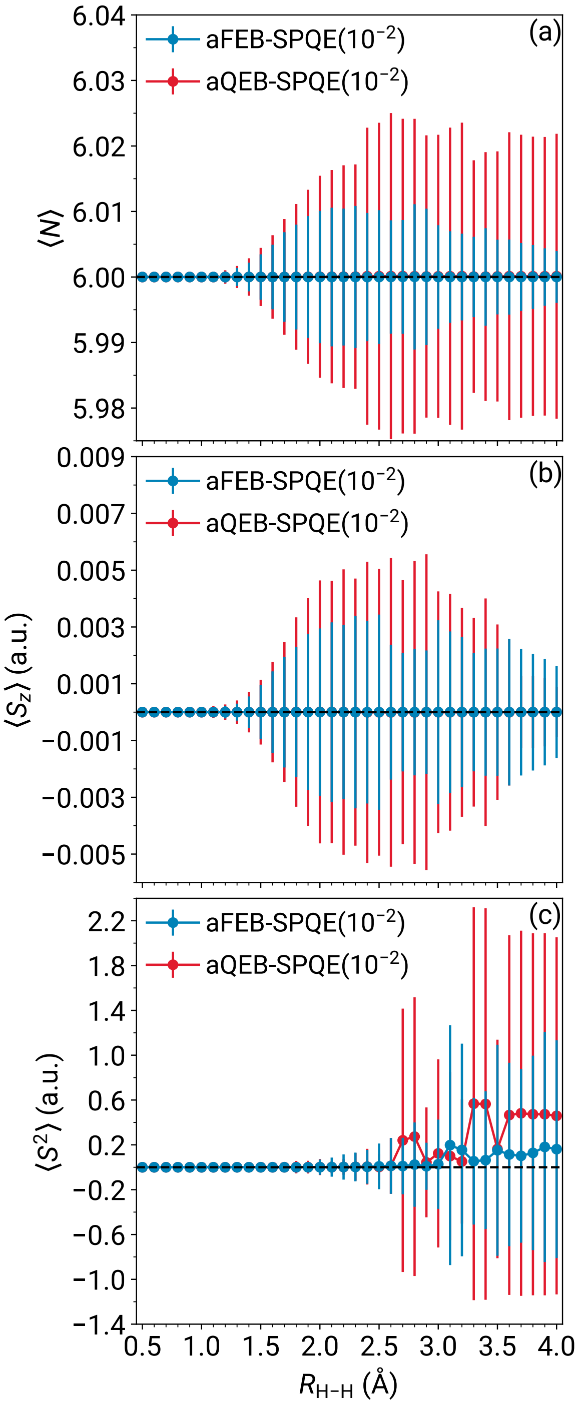


Figure S11: Expectation values of (a) the particle number $N$, (b) the projection of the total spin on the $z$ axis $S_z$, and (c) the total spin squared $S^2$ operators characterizing the aFEB- and aQEB-SPQE($10^{-2}$) simulations of the symmetric dissociation of the linear $H_6$/STO-6G system. The vertical lines denote standard deviations, computed as $\sigma_A = \sqrt{\langle A^2 \rangle - \langle A \rangle^2}$. The horizontal dashed lines denote the corresponding eigenvalues for the ground electronic state of the $H_6$/STO-6G linear chain.

## S2.3 Ring $H_6$/STO-6G: FEB-SPQE vs aFEB-SPQE

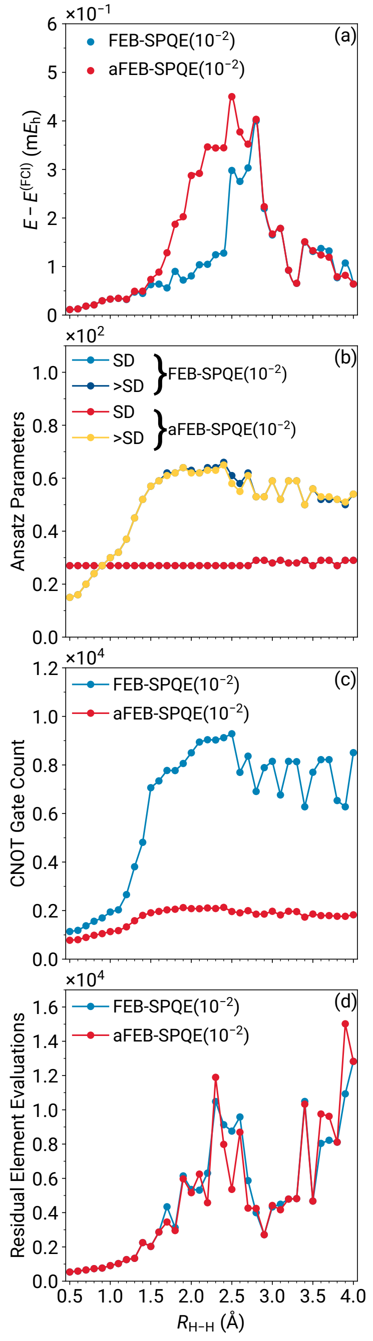


Figure S12: Errors relative to FCI [(a)], ansatz parameters [(b)], CNOT gate counts [(c)], and residual element evaluations [(d)] characterizing the FEB- and aFEB-SPQE($10^{-2}$) simulations of the symmetric dissociation of the $H_6$/STO-6G ring. The "SD" and ">SD" symbols denote single or double (SD) or higher (>SD) excitation operators.

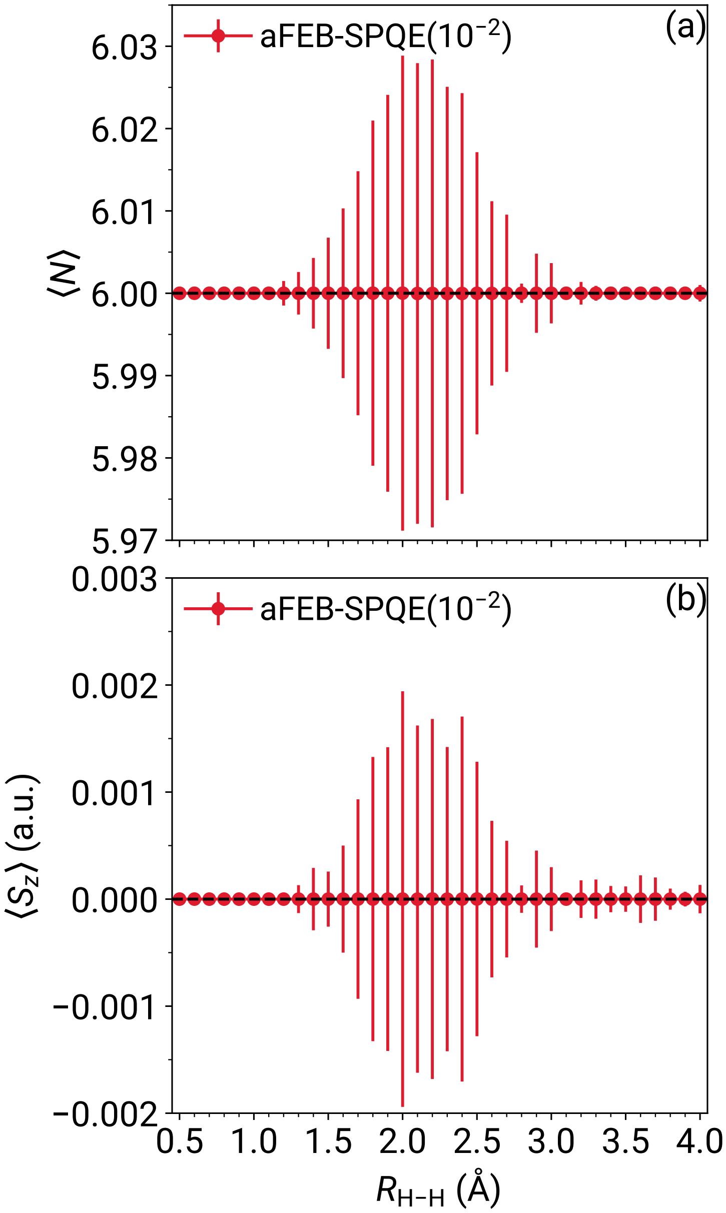


Figure S13: Expectation values of (a) the particle number $N$ and (b) the projection of the total spin on the $z$ axis $S_z$ operators characterizing the aFEB-SPQE($10^{-2}$) simulations of the symmetric dissociation of the $H_6$/STO-6G ring. The vertical lines denote standard deviations, computed as $\sigma_A = \sqrt{\langle A^2 \rangle - \langle A \rangle^2}$. The horizontal dashed lines denote the corresponding eigenvalues for the ground electronic state of the $H_6$/STO-6G ring.

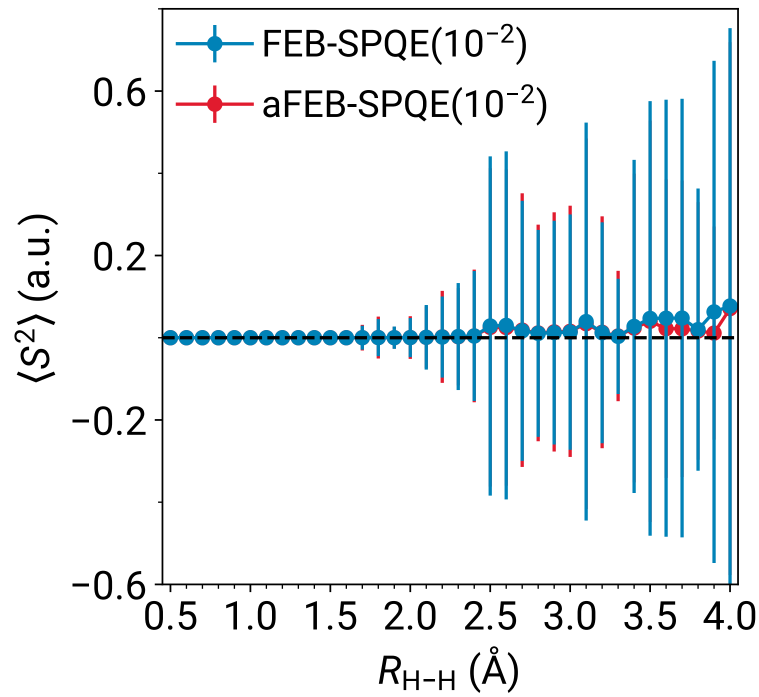


Figure S14: Expectation values of the total spin squared $S^2$ operator characterizing the FEB- and aFEB-SPQE($10^{-2}$) simulations of the symmetric dissociation of the $H_6$/STO-6G ring. The vertical lines denote standard deviations, computed as $\sigma_A = \sqrt{\langle A^2 \rangle - \langle A \rangle^2}$. The horizontal dashed line denotes the corresponding eigenvalue for the ground electronic state of the $H_6$/STO-6G ring.

## S2.4 "Zig-zag" $H_6$/STO-6G: FEB-SPQE vs aFEB-SPQE

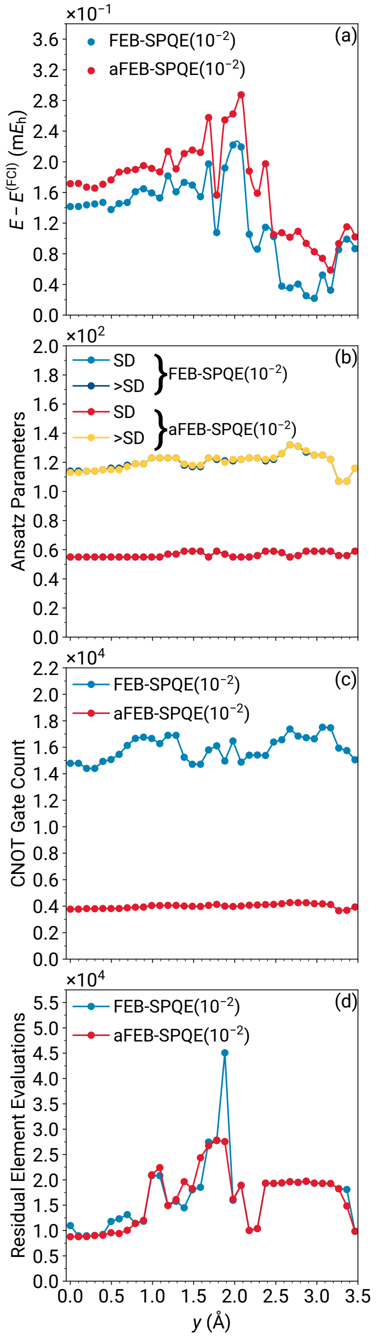


Figure S15: Errors relative to FCI [(a)], ansatz parameters [(b)], CNOT gate counts [(c)], and residual element evaluations [(d)] characterizing the FEB- and aFEB-SPQE($10^{-2}$) simulations of the $C_{2h}$-symmetric dissociation of the "zig-zag" $H_6$/STO-6G system. The "SD" and ">SD" symbols denote single or double (SD) or higher (>SD) excitation operators.

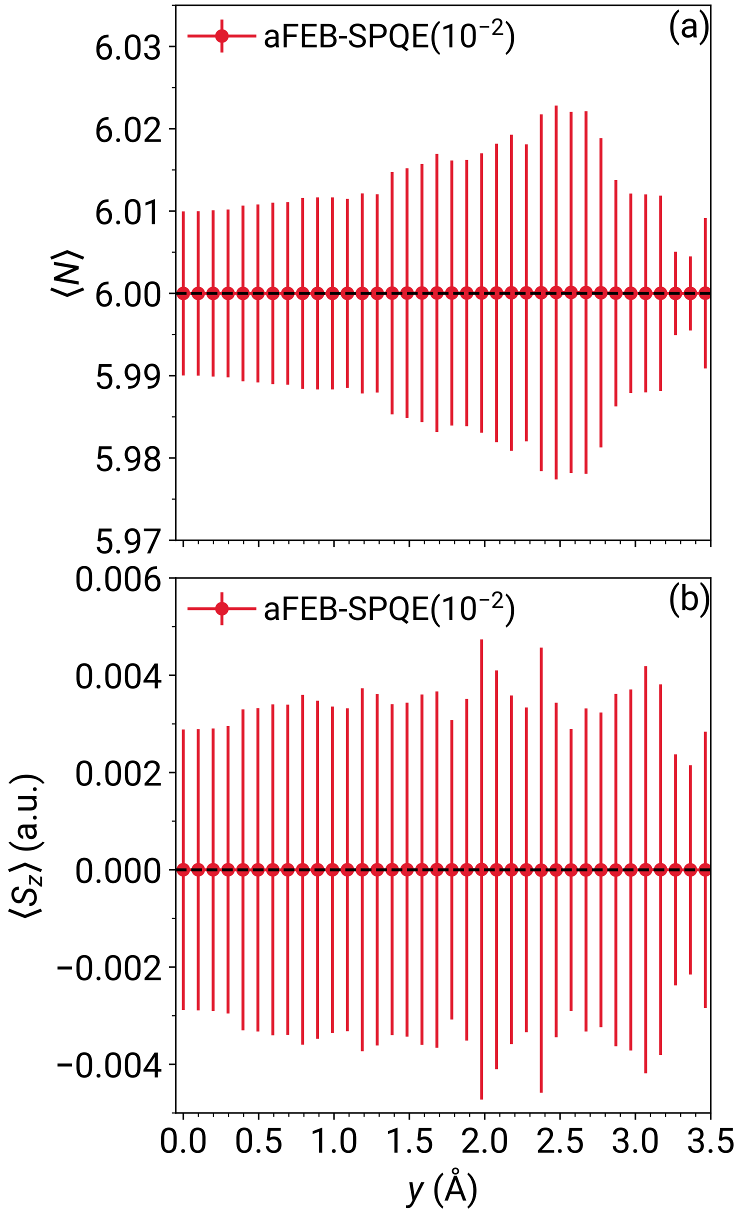


Figure S16: Expectation values of (a) the particle number $N$ and (b) the projection of the total spin on the $z$ axis $S_z$ operators characterizing the aFEB-SPQE($10^{-2}$) simulations of the $C_{2\text{h}}$-symmetric dissociation of the "zig-zag" $H_6$/STO-6G system. The vertical lines denote standard deviations, computed as $\sigma_A = \sqrt{\langle A^2\rangle - \langle A\rangle^2}$. The horizontal dashed lines denote the corresponding eigenvalues for the ground electronic state of the "zig-zag" $H_6$/STO-6G system.

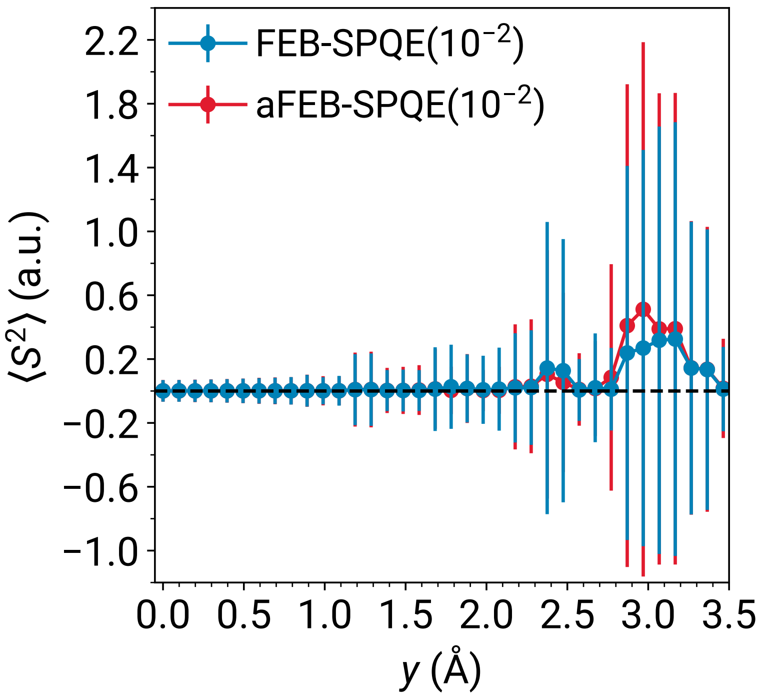


Figure S17: Expectation values of the total spin squared $S^2$ operator characterizing the FEB- and aFEB-SPQE($10^{-2}$) simulations of the $C_{2\mathrm{h}}$-symmetric dissociation of the "zig-zag" $H_6$/STO-6G system. The vertical lines denote standard deviations, computed as $\sigma_A = \sqrt{\langle A^2 \rangle - \langle A \rangle^2}$. The horizontal dashed line denotes the corresponding eigenvalue for the ground electronic state of the "zig-zag" $H_6$/STO-6G system.
